# Safety and effectiveness of vinflunine in patients with metastatic transitional cell carcinoma of the urothelial tract after failure of one platinum-based systemic therapy in clinical practice

**DOI:** 10.1186/1471-2407-14-779

**Published:** 2014-10-24

**Authors:** Daniel Castellano, Javier Puente, Guillermo de Velasco, Isabel Chirivella, Pilar López-Criado, Nicolás Mohedano, Ovidio Fernández, Icíar García-Carbonero, María Belén González, Enrique Grande

**Affiliations:** Medical Oncology Department, 12 de Octubre University Hospital, Madrid, Spain; Medical Oncology Department, Clínico San Carlos University Hospital, Madrid, Spain; Research Fellow in Uro-Oncology, Cambridge University Health Partners, Cambridge, UK; Medical Oncology Department, Clínico de Valencia University Hospital, Valencia, Spain; Medical Oncology Department, MD Anderson Cancer Center, Madrid, Spain; Medical Oncology Department, Guadalajara University Hospital, Guadalajara, Spain; Medical Oncology Department, Orense Hospital Complex, Orense, Spain; Medical Oncology Department, Virgen de la Salud University Hospital, Toledo, Spain; Medical Oncology Department, Son Llatzer Hospital, Mallorca, Spain; Medical Oncology Department, Ramón y Cajal University Hospital, Ctra. de Colmenar Viejo km. 9,100, 28034 Madrid, Spain

**Keywords:** Activity, Community setting, Second-line, Urothelial carcinoma, Vinflunine

## Abstract

**Background:**

Patients with transitional cell carcinoma of the urothelial tract (TCCU) who fail initial platinum-based chemotherapy for advanced disease represent a challenge in daily clinical practice. Vinflunine is approved by the European Medicine Agency (EMA) but, up to now, limited experience has been reported outside clinical trials.

**Methods:**

We assessed the efficacy and safety of vinflunine in an unselected group of 102 consecutive patients with metastatic TCCU.

**Results:**

The median age was 67 years (range 45–83). Among the most common comorbidities that patients presented at baseline were hypertension (50.5%) and diabetes (20.7%).

Distant metastases were present in retroperitoneal nodes (58%), lung (29.3%), and bone (20.2%). The ECOG 0, 1 and 2 performance status at the start of vinflunine were 31.3%, 60.6% and 8.1%, respectively. The most commonly reported adverse events of any grade were constipation 70.6% (5.9% grade 3–4), vomiting 49.1% (2% grade 3–4), neutropenia 48.1% (12.8% grade 3–4) and abdominal pain 34.3% (4.9% grade 3–4). A median of 4 cycles of vinflunine was administered per patient (range 1–18). Median progression free and overall survival for all patients (N = 102) were 3.9 months (2.3-5.5) and 10 months (7.3-12.8), respectively. Time to tumor progression was 4.3 months (2.6-5.9). Two patients (2%) achieved CR, 23 (22.5%) patients had PR, and 42 (41.2%) presented SD as best response. The clinical benefit rate with vinflunine was 65.7%.

**Conclusions:**

Our results show that the behavior of vinflunine in routine clinical practice resembles that of the pivotal phase III randomized study.

## Background

Transitional cell cancer of the urothelial tract (TCCU) represents a major health problem worldwide. In fact, TCCUs are the sixth most common type of cancer in western countries [1]. Traditionally, advanced TCCUs have been considered chemosensitive tumors based on high radiological response rates of 40-70% with cisplatin-based schemes such as gemcitabine-cisplatin (GC), methotrexate, vinblastine, doxorubicin, and cisplatin (M-VAC) or paclitaxel, cisplatin, and gemcitabine (PCG) [2–4]. Unfortunately, responses are not maintained over time and median progression free and overall survivals rarely exceed 8 and 15 months, respectively, when metastatic TCCU patients are treated in first-line [5–8]. Patients who fail the initial systemic approach for advanced disease represent a challenge in daily clinical practice.

In the last decade, wide ranges of single agents or combination schemes have been tested for activity in patients who are resistant to previous platinum approaches. The drugs explored in this setting included paclitaxel, [9] nab-paclitaxel, [10] irinotecan, [11] ixabepilone, [12] bortezomib, [13] pemetrexed, [14] oxaliplatin, [15] ifosfamide, [16] lapatinib, [17] docetaxel, [18] gemcitabine, [19] topotecan, [20] gefitinib, [21] sorafenib, [22] sunitinib, [23] and pazopanib [24]. The most promising combined chemotherapy schemes among those studied were paclitaxel plus gemcitabine, [25] ifosfamide plus gemcitabine [26] or carboplatin plus paclitaxel [27]. Despite the great efforts and resources devoted to all these trials, together with the number of patients involved, in most cases the clinical outcomes were disappointing with objective response rates ranging between 10 and 20%, median progression free survivals of 2–3 months, and median overall survivals of 6–9 months [28].

Vinflunine is the newest member of the vinca alkaloids family available to clinical practice [29]. As with other tubulin inhibitors, vinflunine prevents microtubule assembly during mitosis and induces apoptosis [30, 31]. The main differentiating feature that distinguishes vinflunine from others vinca alkaloids is the affinity profile of vinflunine which has a greater effect on mitotic rather than axonal tubulin. Therefore, the result is a significantly reduced rate of neurotoxicity which allows for greater plasma concentrations of the drug [32]. The clinical activity of vinflunine in patients with metastatic TCCU was initially assessed in two non-randomized phase II trials [33, 34]. The earlier phase II trials showed that the activity of vinflunine in 51 and 175 platinum-resistant TCCU patients achieved response rates of 18% and 15%, respectively, and median duration of responses were 9.1 and 6 months. Median progression free survival and overall survival were 3.0 and 6.6 months in the first trial, and 2.8 and 8.2 months in the second one. These consistent results led to a pivotal, multinational, and randomized study that compared vinflunine and best supportive care in second-line treatment of advanced TCCU patients who had previously progressed after a platinum-containing regimen [35]. A total of 370 patients were recruited and vinflunine was shown to be superior to the control arm in terms of the considered primary endpoint of the study which was overall survival in the intention to treat population (6.9 months vs. 4.6 months). However, these results were not found to be statistically significant (HR 0.88; 95% CI, 0.69-1.12: P = 0.287).

All others efficacy parameters favored vinflunine clinically and were statistically significant, such as overall survival in the analysis per protocol population (6.9 vs. 4.3 months: P = 0.04), overall response rate (16% vs 0%: P = 0.0063), disease control rate (41.1% vs 24.8%: P = 0.0024), and median progression free survival (3.0 months vs 1.5 months: P = 0.0012). The duration of objective responses was 7.4 months (95% CI 4.5 to 17.0 months) in those patients treated with vinflunine. Long-term overall survival data from this registration trial after a follow-up of more than 45 months confirmed the increase in total median overall survival with vinflunine compared to best supportive care in the intention to treat population (6.9 months vs. 4.6 months) and the statistically significant increase in the eligible population (6.9 vs. 4.3 months; HR 0.78; 95% CI 0.61-0.96: P = 0.00227) [36]. As a result of this study, vinflunine was the first drug to receive approval from the European Medicine Agency (EMA) for use in platinum-resistant metastatic TCCU patients. We conducted a retrospective, observational, and non interventional study (according to the classification of the Spanish Health Authorities) to assess the impact of treatment with vinflunine in our daily practice in terms of toxicity, response rate, duration of response, progression free survival, and overall survival in an unselected subgroup of patients with metastatic TCCU who had progressed after only one previous line of platinum-containing regimen for advanced disease, and furthermore assessed the reproducibility of the clinical trial results in routine clinical practice.

## Methods

One hundred and two consecutive outpatients with metastatic TCCU who were treated with vinflunine in 15 university and community hospitals spread all along Spain were analyzed for safety and activity. Patients started treatment between December 2009 and June 2013, and follow up and dose adjustments were performed according to local investigators criteria. A normalized database with uniform CRF’s adapted to urothelial cancer features, was prepared for the data collection. Data were entered into databases by the own investigators. Concerning to the eligible population, it included adult patients with advanced TCCU who had previously failed to one prior first-line regimen based on platinum. All patients were offered for systemic treatment with vinflunine for the advanced disease under approved conditions. Dose delays and dose modifications were accepted according to the vinflunine package insert. All patients signed the correspondent inform consent in accordance to good clinical practices and local authorities regulation. The study was submitted for validation in the Ethic Committee Hospital 12 de Octubre as stated in the Royal Decree 223/2004 and Article 58 of Law 29/2006 for Post Authorization Retrospective studies. Moreover, it’s a clinical routine practice the use of an Inform Consent for the patient to receive chemotherapy and the use of their data under the personal data protection as detailed in the Spanish Data Protection Law; Organic Law 15/1999 of December 13rd on the protection of personal data.

Patients were deemed ineligible for the analysis if they had received more than one previous chemotherapy regimen for metastatic disease or had received any non-approved chemotherapy agent after failure of a platinum-based scheme. The study included the following demographic variables: gender, age, prior cisplatin or carboplatin regimens, response to first-line treatment approach, comorbidities, primary tumor location, pathological details, surgery performed for the primary tumor, and metastasis location. Objective clinical response [complete response (CR), partial response (PR), stable disease (SD), or progressive disease (PD)] was evaluated by Response Evaluation Criteria in Solid Tumors (RECIST) version 1.1 through computed tomography (CT) scans. To evaluate tumor response to treatment, thoracoabdominal CT scans were performed according to the investigator’s routine clinical practice. Univariate analyses of overall survival and progression free survival were carried out using the Kaplan Meier method, with test of statistical significance performed using the log-rank test with 95% confidence intervals.

Progression free survival was measured from the date of consent to the use of vinflunine to either the date of first objective evidence of disease progression or date of death, whichever occurred first. Overall survival was measured from the date of consent to the use of vinflunine to the date of death from any cause. Estimates of hazard ratios were obtained using the Cox proportional hazards model. Tests of statistical significance were carried out at the 5% two-sided significance levels. All statistical analyses were performed using the SPSS for Windows software package (Rel. SPSS 14.0; SPSS Chicago, IL). The disease control rate was defined as the percentage of patients who had a best response rating of CR, PR, or SD.

Safety involved carrying out a toxicity assessment every time a patient visited an investigators’ clinic or emergency room. Adverse events were reported according to the National Cancer Institute’s (NCI) Common Terminology Criteria for Adverse Events (CTC AE; version 4.0).

## Results

One hundred and two metastatic TCCU patients who had previously failed one prior platinum-containing systemic therapy were treated with vinflunine as a single agent in a second-line setting. Baseline characteristics are listed in Table 1. The median age was 67 years (range 45–83) and the majority of patients had the primary tumor located in the bladder (83.2%). Among the most common comorbidities that patients presented at baseline were hypertension (50.5%) and diabetes (20.7%). The majority of patients had urothelial carcinoma histology (88.2%) with a high degree of histological differentiation (86.9%). Up to 73.5% of patients required at least one previous transurethral resection for localized primary lesions. The most common locations for distant metastasis were the retroperitoneal nodes (58%) followed by lung metastasis (29.3%) and bone metastasis (20.2%). When beginning vinflunine, 60% of patients had a performance status of ECOG 1. Additionally, 31.3% and 8.1% of the patients presented ECOG 0 or 2, respectively. Previous platinum-based treatment in first-line was split between cisplatin (47%) and carboplatin (51%). Two patients had received paclitaxel plus gemcitabine as first-line treatment. A total of 57.9% had achieved either PR or CR with a previous platinum-based chemotherapy. All patients were included for the assessment of toxicity. The most commonly reported adverse events of any grade were constipation 70.6% (5.9% grade 3–4), vomiting 49.1% (2% grade 3–4), neutropenia 48.1% (12.8% grade 3–4) and abdominal pain 34.3% (4.9% grade 3–4) (Table 2). No toxicity-related deaths were reported.

**Table 1:** **Baseline characteristics of the study population**

Characteristics	Total, N = 102 (%)
Age, y	
Median	67
Range	45-83
Primary tumor location	
Bladder	84 (82.4%)
Renal pelvis	11 (10.8%)
Ureter	4 (3.9%)
Prostatic urethra	1 (1.0%)
Ureter + Renal pelvis	1 (1.0%)
Patients comorbidities	
Hypertension	50.5%
Diabetes	20.7%
Lipid metabolism alterations	14.4%
Heart medical records	7.8%
Lung diseases	13.3%
Location of distant metastasis	
Retroperitoneal nodes	58 (58.0%)
Lung	29 (29.3%)
Bone	20 (20.2%)
Liver	17 (17.2%)
ECOG performance status when starting vinflunine	
0	31 (31.3%)
1	60 (60.6%)
2	8 (8.1%)
Prior platinum Regimen	
Cisplatin	47 (47%)
Carboplatin	52 (51%)
Paclitaxel/gemcitabine	2 (2%)
Unknown	1 (1%)

**Table 2:** **Adverse events that occurred in more than 10% of 102 patients treated with vinflunine**

Adverse event	All treated patients N = 102, N (%)	
	All grades	Grade 3/4
Constipation	72 (70.6%)	6 (5.9%)
Vomiting	50 (49.1%)	2 (2.0%)
Neutropenia	49 (48.1%)	13 (12.8%)
Abdominal pain	35 (34.3%)	5 (4.9%)

A median of 4 cycles of vinflunine was administered per patient (range, 1 cycle). The patients received an initial dose of 320 mg/m2, 280 mg/m2 or 250 mg/m2 of vinflunine according to the summary of product characteristics. 32 patients (31.37%), 43 patients (42.16%), and 12 patients (11.76%) of the patients received 320 mg/m2, 280 mg/m2 or 250 mg/m2 of vinflunine respectively.

**Figure 1:**
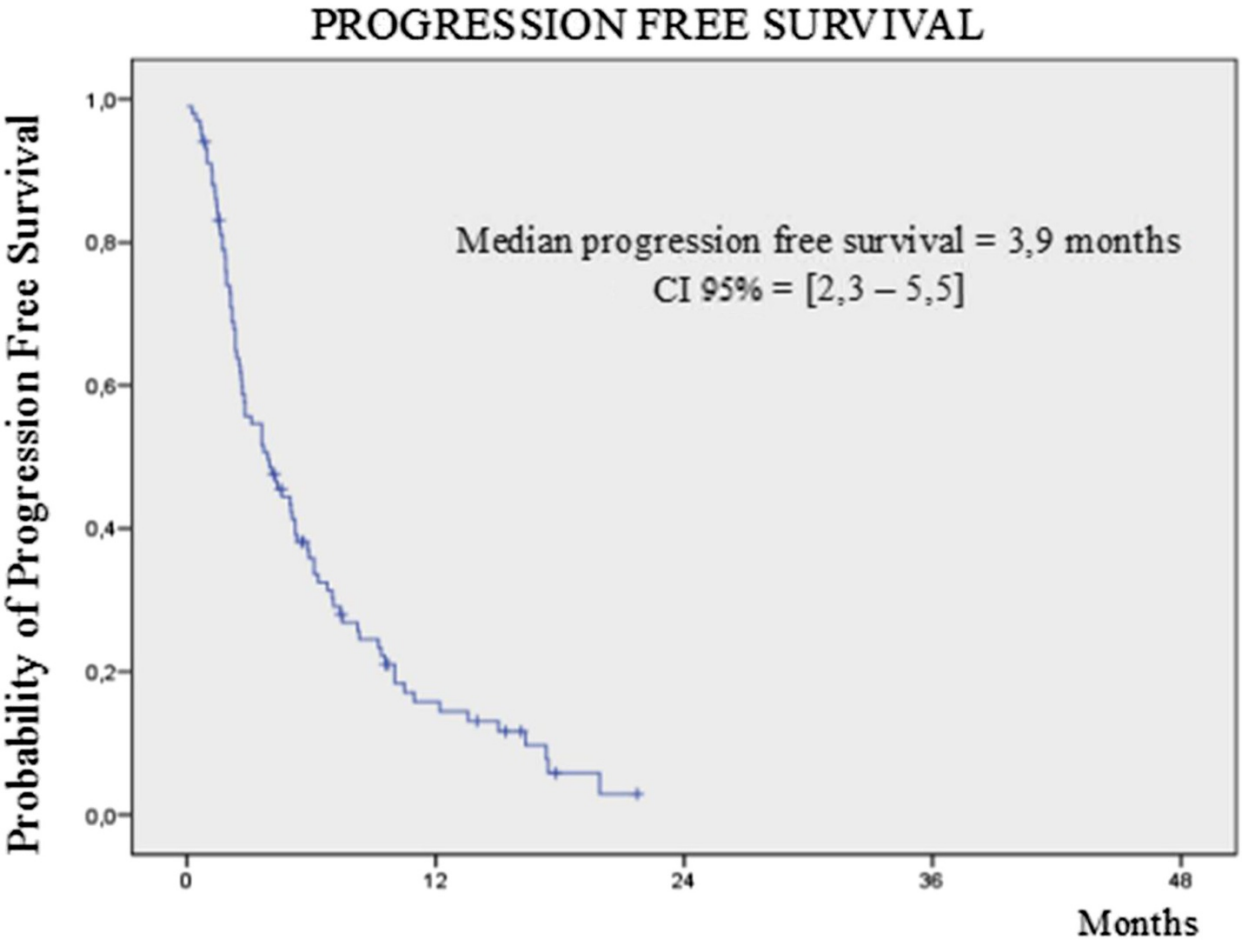
**Kaplan Meier curve by local assessment of progression free survival for the 102 patients with platinum resistant TCCU treated with vinflunine.**

**Figure 2:**
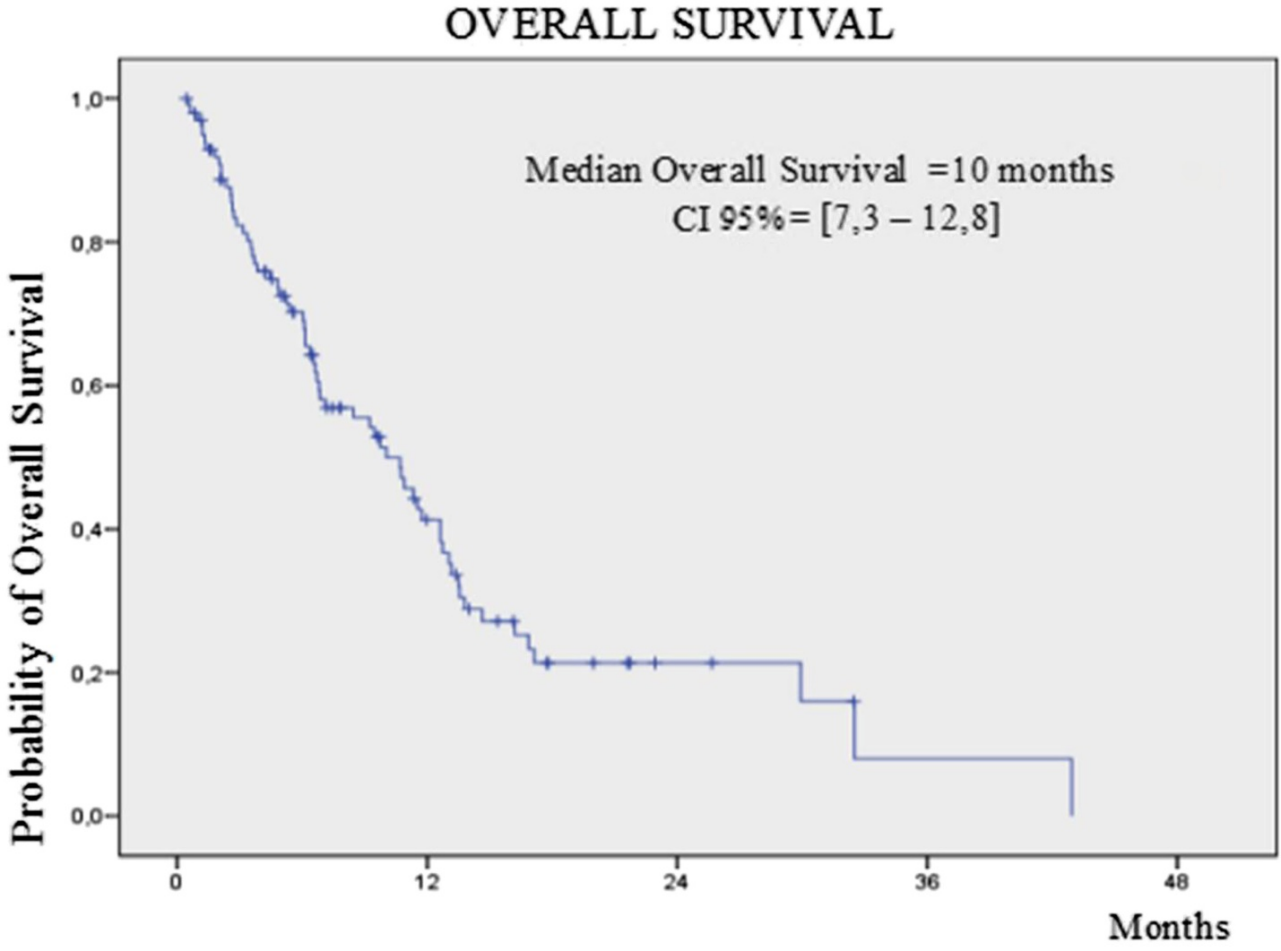
**Kaplan Meier curve by local assessment of overall survival for the 102 patients with platinum resistant TCCU treated with vinflunine.**

After a median follow up of 8.9 months, 81 (79.4%) patients had progressive disease and 66 (64.7%) had died by any cause. Median progression free and overall survival for all patients (N = 102) was 3.9 months (2.3-5.5) and 10 months (7.3-12.8), respectively (Figures 1 and 2). Time to tumor progression in the whole population was 4.3 months (2.6-5.9). Radiological response was evaluable in 98 patients. Two patients (2%) achieved a CR, 23 (22.5%) patients had PR, and 42 (41.2%) presented SD as best response. The clinical benefit rate with vinflunine in the intent to treat populations was 65.7% of those treated (Table 3).

**Table 3:** **Overall Response Rates according to the investigators**

Best response	Investigator assessment N (%)
Complete response	2 (2.0%)
Partial response	23 (22.5%)
Stable disease	42 (41.2%)
Progressive disease	31 (30.4%)
Not evaluable	4 (3.9%)
Overall tumor response rate (CR + PR)	25 (24.5%)
Clinical benefit rate (CR + PR + SD)	67 (65.7%)

Median duration of response for those patients who achieved a complete or partial response (25 patients) was 9.6 months (CI 95% 6.7 – 12.4 months). Median progression free survival for those patients with stable disease (42 patients) as best response by RECIST was 5 months (CI 95% 3.7-6.4 months).

**Figure 3:**
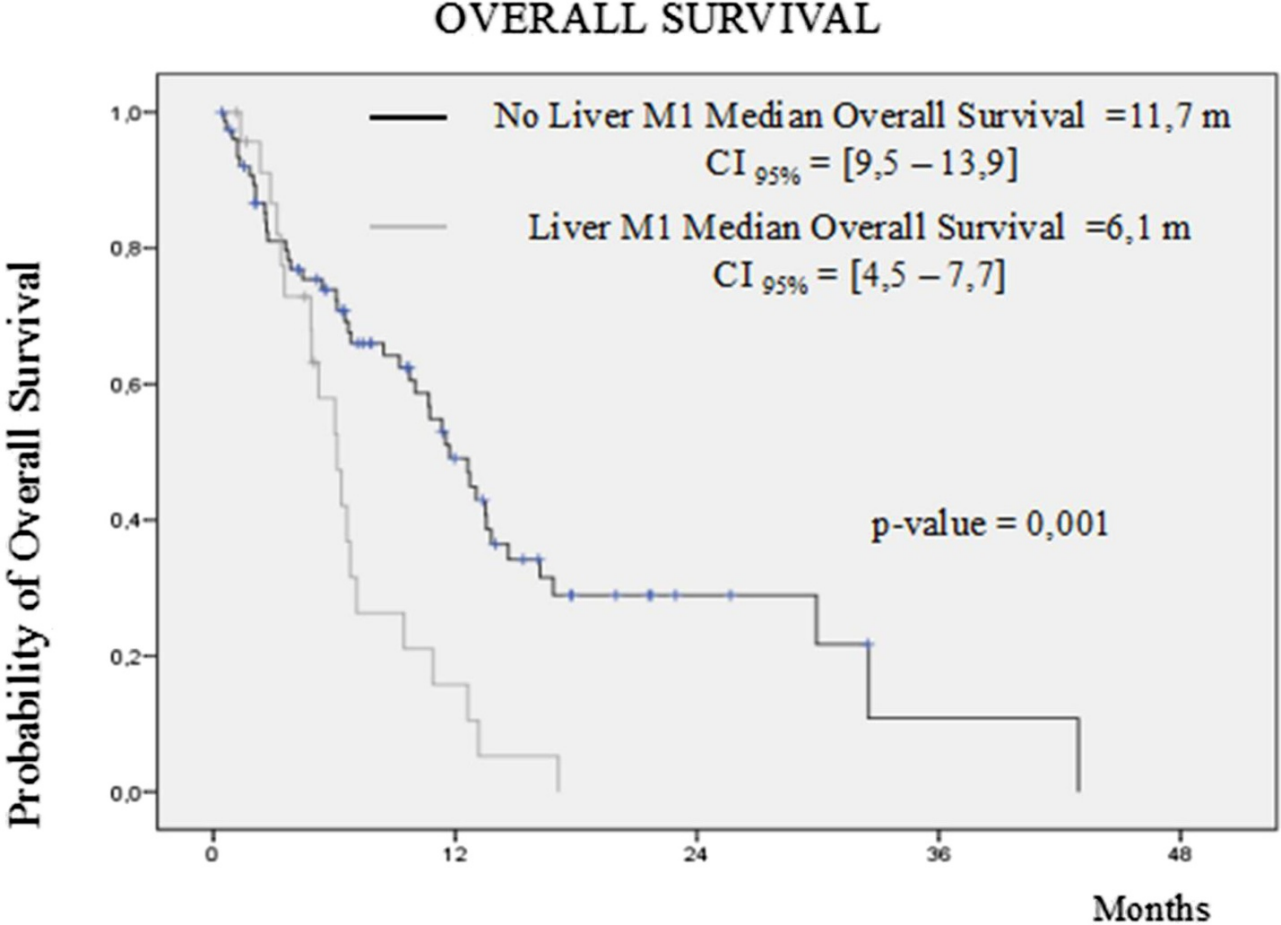
**Kaplan Meier curve by local assessment of overall survival according to the presence of liver metastases.**

**Figure 4:**
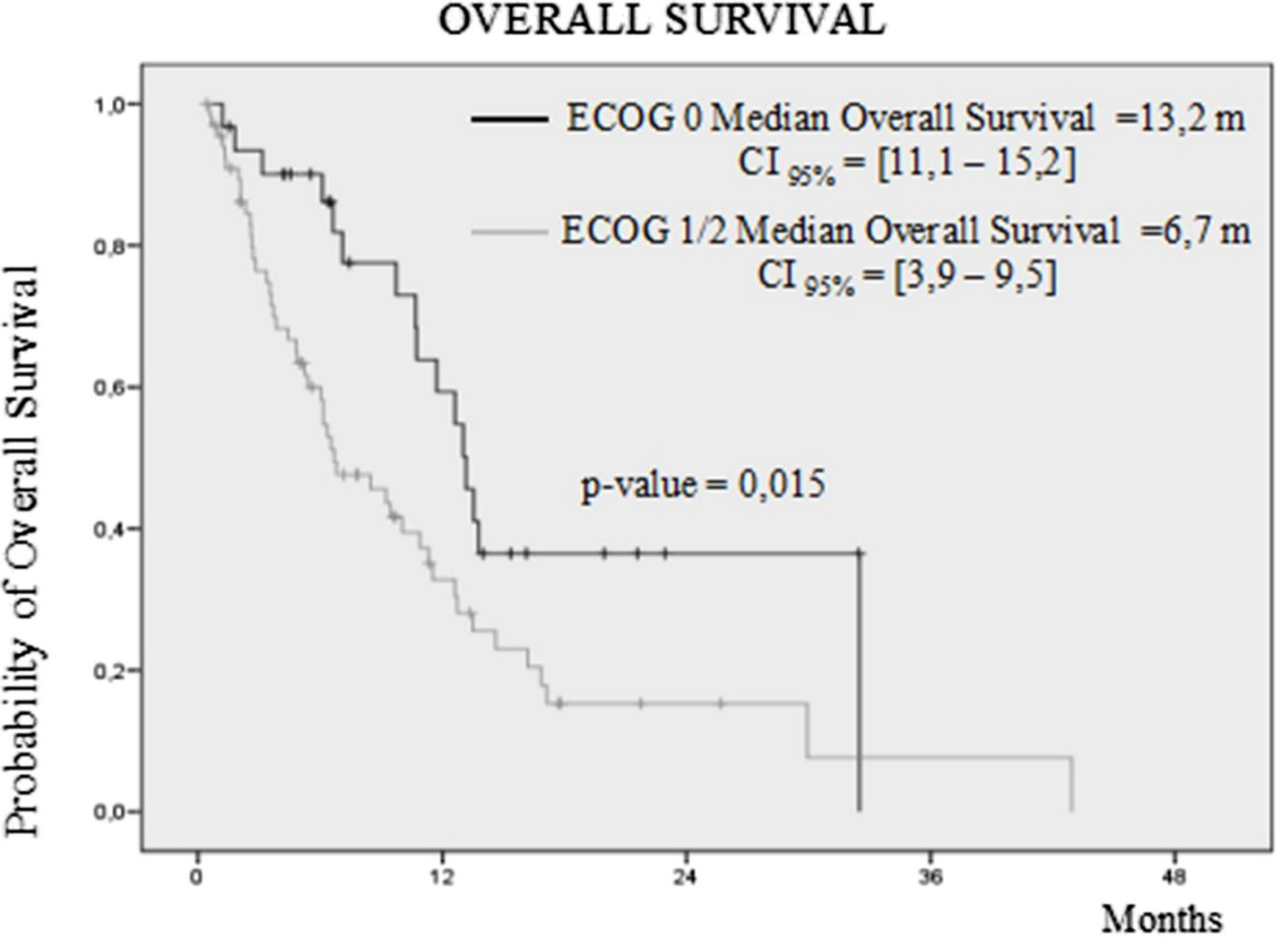
**Kaplan Meier curve by local assessment of overall survival according to ECOG performance status at start of vinflunine.**

Among those with ECOG 0 or 1, median progression free survival was 7.0 (2.8-11.1) months and 3.1 (2.0-4.2) months, respectively (HR = 1.49; 95% CI 0.92-2.41: p = 0.102). Those patients with liver metastatic involvement (17.2%) had a median progression free and overall survival of 2.3 and 6.1 months, respectively (Figure 3). By contrast, all patients without liver metastasis (82.8%) had a median progression free survival of 4.4 months and overall survival of 11.7 months. On the other hand, those patients asymptomatic at start of vinflunine (ECOG 0) had an overall survival of 13.2 months that compared favorably with the overall survival achieved by those patients with ECOG 1 or 2 (6.7 months) (Figure 4).

## Discussion

The disseminated platinum-resistant TCCU population is clearly an unmet clinical need which, up to now, has not been solved. Modest activity in terms of overall survival in the pivotal trial means that patients’ access to this drug might not be as fluid as it should. Therefore, a high proportion of patients with advanced TCCU will not receive any active treatment after failure of prior platinum-containing schemes.

Our study demonstrates that vinflunine is an active and safe drug for patients with platinum-resistant urothelial carcinomas treated in routine daily practice. The efficacy that we obtained was similar to the results achieved in the registration trial. In this respect, we reported an overall response rate of 24.5% which compares more than favorably with that achieved in the registration trial (8.1%). Disease control rate was also greater in our study (65.7%) than in the pivotal trial (41.1%). Two (2%) of the patients that received second-line vinflunine for advanced TCCU achieved a confirmed CR.

Similar results were achieved in terms of progression free and overall survival; in our series we observed 3.9 months and 10 months, respectively, which compared favorably to the 3.0 and 6.9 months achieved in the registration trial. Moreover, we were able to administer more cycles of vinflunine (4) to our patients than were administered to those in the pivotal trial (3). This is of particular importance since it reflects the good safety profile and management of vinflunine in daily clinical practice where comorbidities and patient performance status are very different to those in the selected patients recruited for industry-sponsored trials. To support the concept of vinflunine’s good tolerability in daily clinical practice we can compare several adverse events grade 3 or 4 with those observed in the pivotal trial, such as constipation (5.9% vs 16.1%), vomiting (2% vs 2.8%), neutropenia (12.8% vs 50%), and abdominal pain (4.9% vs 4.0%). Nevertheless, since the image evaluation techniques were performed according to local practices, there could have been a delay in the evaluation timeline of responses in comparison with the fixed timeframe executed in the pivotal trial. This fact may affect the duration of vinflunine treatment in our series.

The presence of visceral metastasis, a poor performance status (ECOG > 0), and low basal hemoglobin levels (<10 gr/dl) are considered poor prognostic factors for overall survival in patients with metastatic TCCU who experienced treatment failure with the first-line platinum-based regimen included in the phase III vinflunine trial [37, 38].

We also saw that impact on prognosis in our patients: those patients with ECOG 1 or 2 had less progression free and overall survival than those with ECOG 0 at baseline (3.1 months and 6.7 months vs 7.0 months and 13.2 months). The same was found with respect to the presence of visceral metastasis at the time of entering into the study. Those patients who had visceral metastasis in lungs or liver had poorer median progression free and overall survivals (2.6 months vs 7.1 months) than those who only had lymph nodes and/or bone metastasis involvement (6.1 months vs 11.3 months).

Similarly, patients with liver involvement had a statistically significant worst progression free survival (2.3 vs. 4.4 months; HR 1.66 CI95% 1.02-2.7: p = 0.039) and overall survival (6.1 vs. 11.7 months; HR 2.44 CI 95% 1.41-4.23: p = 0.001) than those without liver involvement.

We could not find so far any correlation between initial dose used with clinical outcome nor a need for a further dose reduction.

The present study has several design limitations, such as being a retrospective analysis with no control group, efficacy and safety monitoring were not pre-specified (the investigator’s own practice) and the bias in the selection of patients who were candidates for a second-line treatment approach, among others. However, the elevated rate of baseline comorbidities of patients included in the study, the high rate of distant metastasis and the high proportion of ECOG >0 make this group a true reflection of the general population of metastatic TCCU patients. Nevertheless, we lacked the candidate biomarkers of prospectively designed vinflunine trials which may have allowed us to better select patients who would have had greater possibilities of a higher clinical benefit. Along these lines, the Spanish Oncology Genitourinary Group (SOGUG) cooperative group is currently exploring the correlation between efficacy, antiangiogenic tissue markers and epithelial-mesenchymal transition tissue markers in those patients who gave special informed consent and were treated with vinflunine.

Two similar experiences of vinflunine use in daily routine practice in Germany and France were recently reported in an abstract form [39, 40]. German colleagues reported a response rate of 23.4% with a median overall survival of 7.7 months and an average of 4.7 cycles administrated to 77 TCCU patients. French colleagues reported the results from 134 patients who received a median of 5 (1 to 23) cycles to reach a median progression free survival of 4.2 months, an overall response rate of 22% and an overall survival of 8.2 months. Cross report comparison seems to confirm that the outcomes achieved in the registration trial are reproducible in routine daily practice.

In conclusion, the results from this study confirm that the efficacy and safety of vinflunine in second-line treatment of metastatic TCCU patients who have failed platinum-based schemes in a trial population can be reproduced in an unselected group of patients with metastatic TCCU. Compared to published data, those patients treated with vinflunine in daily clinical practice show similar results to those previously reported. The patients included in our study represent an unselected group with metastatic TCCU and, therefore, the results demonstrate that the pivotal results can be reproduced in the general population. No relevant differences in toxicity patterns and length of treatment were observed. These results are encouraging and imply that the clinical trial results seen with vinflunine could be translated into routine clinical practice. Taking these results into consideration, vinflunine seems to be a reasonable option in daily clinical practice for patients with advanced TCCU who experience progression after first-line platinum-based chemotherapy.

## Conclusions

Patients who progress under or after platinum-based chemotherapy schemes have a very poor prognosis with a life expectancy of less than 6 months. Vinflunine is the only drug which has been approved, at least in Europe, for the treatment of these patients. Clinical setting reproducibility of the outcomes achieved in pivotal clinical trials is sometimes troublesome. We performed a retrospective analysis of the clinical outcome in terms of activity and safety in 102 unselected, consecutively treated patients with metastatic TCCU who had previously progressed after one prior platinum-containing regimen. Patients received a median of 4 cycles of vinflunine treatment (range between 1 and 18). Median progression free and overall survival for all patients (N = 102) was 3.9 months (2.3-5.5) and 10 months (7.3-12.8), respectively. Time to tumor progression in the intention to treat population was 4.3 months (2.6-5.9). Radiological response was evaluable in 98 patients. Two patients (2%) achieved a CR, 23 (22.5%) patients had PR, and 42 (41.2%) presented SD as best response. Furthermore, 65.7% of patients demonstrated a clinical benefit with vinflunine. These results of vinflunine in daily clinical practice resemble those achieved in the pivotal trial. The toxicity profile was also similar to that reported previously. Taking all these outcomes into consideration we believe that the results are encouraging and imply that the clinical trial results obtained with vinflunine can be translated into routine clinical practice. Nevertheless, there is an overwhelming need to incorporate new objective translational biomarkers that might help us better select the right treatment for our patients.

## Authors’ information

Daniel Castellano is the first author.
